# Supplementary material for: Discovering sparse transcription factor codes for cell states and state transitions during development
Source: eLife. 2017 Mar 15;6:e20488. doi: 10.7554/eLife.20488 (PMC5352226; doi:10.7554/eLife.20488)
Supplement: Figure 2—source data 1. — Listed for each early hematopoietic progenitor whose name is abbreviated in this paper are the Immunological Genome Project descriptor for the cell type, its common name and phenotype, its age and location, and the number of replicates in the data set. DOI: http://dx.doi.org/10.7554/eLife.20488.009 [file elife-20488-fig2-data1.docx]

| Abbreviation in text | Immgen Descriptor | Long name | Phenotype | Age | Location | Number of replicates |
| --- | --- | --- | --- | --- | --- | --- |
| LT | SC.LT34F.BM | Long-Term reconstituting Stem Cell (LT-HSC) | CD34- Flk2- Lin- ckit+ Sca1+ | 8w | Bone marrow | 3 |
| ST | SC.ST34F.BM | Multipotent Progenitor (ST-HSC) | CD34+ Flk2- Lin- ckit+ Sca1+ | 8w | Bone marrow | 2 |
| MPP | SC.MPP34F.BM | Multipotent Progenitor (MPP) | CD34+ Flk2+ Lin- ckit+ Sca1+ | 8w | Bone marrow | 2 |
| MLP | MLP.BM | Multilineage Progenitor | Lin- AA4+ Kit++ IL7Ra- B220- | 6w | Bone marrow | 4 |
| CMP | SC.CMP.BM | Common Myeloid Progenitor | Lin- IL7R- Sca1- ckit+ FcgRloCD34+ | 8w | Bone marrow | 2 |
| MEP | SC.MEP.BM | Megakaryocyte-Erythroid Progenitor | Lin- IL7R- Sca1- ckit+ FcgRloCD34- | 8w | Bone marrow | 2 |
| GMP | SC.GMP.BM | Granulocyte-Monocyte Progenitor | Lin- IL7R- Sca1- ckit+ FcgRhiCD34+ | 8w | Bone marrow | 3 |
| CLP | proB.CLP.BM | Common Lymphoid Progenitor | Lin- AA4+ Kit+ IL7Ra+ B220- | 10w | Bone marrow | 4 |
| FrA | proB.FrA.BM | Fr. A (pre-pro-B) | Lin- AA4+ Kit+ IL7Ra+ B220+ | 10w | Bone marrow | 4 |
| FrBC | proB.FrBC.BM | Fr. B/C (pro-B) | Lin- AA4+IgM-CD19+CD43+HSA+ | 10w | Bone marrow | 3 |
| ETP | preT.ETP.Th | Early T lineage Precursor | 4- 8- 11b- 11c- 19- NK1.1- TCR- 44hi 117hi 25- | 6w | Thymus | 3 |

**Figure 2 – Source Data 1: Early Hematopoietic Cell Types Considered**
