# Supplementary material for: Discovering sparse transcription factor codes for cell states and state transitions during development
Source: eLife. 2017 Mar 15;6:e20488. doi: 10.7554/eLife.20488 (PMC5352226; doi:10.7554/eLife.20488)
Supplement: Figure 3—source data 1. — Table with selected marker genes for early hematopoietic cell types, along with references to published validations of their functional role. Genes known to be effective for reprogramming are shown in bold. DOI: http://dx.doi.org/10.7554/eLife.20488.015 [file elife-20488-fig3-data1.docx]

**Figure 3 – Source Data 1: Marker genes for early hematopoiesis**

| Cell type | Selected marker genes | References |
| --- | --- | --- |
| LT | *Egr1, Fus* | Sugawara et al., 2010; Min et al., 2008 |
| ST | *Mpl* | Solar et al., 1998 |
| CMP | *Srf*, *Zeb2*, *Rbpj*, *Irf8*, ***Pbx1***, ***Mycn*** | Goossens et al., 2011; Kurotaki et al., 2013; Ragu et al., 2010; Robert-Moreno et al., 2005; Tamura et al., 2000; Ficara et al., 2013; Laurenti et al., 2008; Riddell et al., 2014 |
| MPP | *Satb1* | Satoh et al., 2013; |
| MLP | ***Lmo2*** | Riddell et al., 2014; Batta et al., 2014 |
